# Role of Reactive Oxygen Species in Collagen-Induced Platelet Activation and the Protective Effects of Antioxidants

**DOI:** 10.3390/antiox14040497

**Published:** 2025-04-20

**Authors:** Jin-Yi Han, Hideo Utsumi, Han-Young Chung

**Affiliations:** 1 Division of Hepatobiliary and Pancreatic Surgery, Department of Surgery, Keimyung University Dongsan Medical Center, 1035 Dalgubeol-daero, Dalseo-gu, Daegu 42601, Republic of Korea; esr0319@naver.com; 2 Faculty of Pharmaceutical Sciences, Kyushu University, 3-1-1 Maidashi, Higashi-ku, Fukuoka 812-8582, Japan; utsumih@u-shizuoka-ken.ac.jp; 3 Faculty of Pharmaceutical Sciences, Shizuoka University, 52-1 Yada, Suruga-ku, Shizuoka 422-8526, Japan; 4 College of Pharmacy, Chungnam National University, Daejeon 34134, Republic of Korea

**Keywords:** platelet, collagen, in vivo ESR, spin-trapping, thrombosis

## Abstract

Collagen plays a crucial role in platelet activation and thrombosis, yet the underlying mechanisms involving reactive oxygen species (ROS) remain incompletely understood. This study investigated how collagen modulates ROS generation and platelet aggregation both in vitro and in vivo, as well as evaluating the protective effects of antioxidants. In vitro, collagen induced dose-dependent platelet aggregation and increased ROS generation, evidenced by the enhanced EMPO adduct formation detected via electron spin resonance (ESR). In vivo experiments demonstrated that collagen administration significantly accelerated CAT-1 decay, indicating elevated oxidative stress with a transient peak around 1 minute post-treatment. Furthermore, escalating collagen doses correlated with increased ROS generation and reduced survival rates in mice, underscoring collagen’s impact on oxidative stress and thrombosis severity. Importantly, treatment with enzymatic antioxidants (superoxide dismutase, catalase) and non-enzymatic antioxidants (DMTU, Tiron, mannitol) significantly attenuated collagen-induced oxidative stress and improved animal survival. Collectively, these findings elucidate the pivotal role of ROS in collagen-induced platelet activation and thrombosis and highlight antioxidants as promising therapeutic candidates for preventing thrombotic disorders and managing cardiovascular risk.

## 1. Introduction

Platelets, small anucleate cells approximately 2–3 μm in diameter, are essential components in maintaining hemostasis and significantly contribute to thrombosis [1,2]. With around 1 × 10^12^ circulating platelets and an average lifespan of approximately 10 days [3,4], these cells rapidly respond to vascular injury by adhering to exposed extracellular matrix components, becoming activated, and aggregating to form a stable hemostatic plug [5,6]. The delicate balance of platelet activity is crucial; insufficient platelet function leads to excessive bleeding, whereas hyperactive platelets can cause thrombotic events such as myocardial infarction, ischemic stroke, and pulmonary embolism [7,8].

Platelet activation primarily occurs through interactions with collagen and other subendothelial matrix proteins, initiating intracellular signaling cascades that result in shape changes, granule secretion, and aggregation, ultimately stabilizing clot formation [9,10]. Collagen activates platelets through specific surface receptors, notably glycoprotein VI (GPVI) and integrin α2β1. GPVI is crucial for initial signaling responses to collagen [11,12], while integrin α2β1 mainly facilitates firm platelet adhesion to collagen-rich matrices [13]. These interactions activate intracellular signaling pathways involving Src family kinases, spleen tyrosine kinase (Syk), phospholipase C gamma 2 (PLCγ2), and calcium mobilization, enhancing platelet activation [14,15].

Concomitant with receptor-mediated platelet activation, reactive oxygen species (ROS), such as superoxide anions, hydroxyl radicals, and hydrogen peroxide, are generated [16,17]. These ROS serve as crucial secondary messengers that potentiate platelet responses and aggregation [18,19]. Elevated ROS levels are critical contributors to platelet hyperactivation, implicated in various thrombotic pathologies, including acute coronary syndrome, myocardial infarction, and stroke [20,21]. Understanding the precise role and regulatory mechanisms of ROS in platelet physiology is essential for developing effective therapeutic strategies aimed at mitigating thrombotic risk [22,23].

Despite substantial advancements in platelet biology research, detailed molecular mechanisms underlying collagen-induced platelet activation, particularly involving ROS-mediated signaling, remain incompletely elucidated [24,25]. Emerging evidence underscores the role of ROS-generating enzymes such as NADPH oxidase, xanthine oxidase, and lipoxygenases, highlighting their involvement in cardiovascular diseases such as atherosclerosis, hypertension, and ischemia–reperfusion injury [26,27]. Studies suggest enzymatic and non-enzymatic antioxidants might effectively attenuate ROS generation, thereby diminishing platelet hyperactivation and reducing thrombotic complications [28,29]. Nevertheless, the direct detection and the detailed characterization of ROS in physiological conditions remain challenging due to their transient nature, high reactivity, and methodological limitations.

Electron spin resonance (ESR) spectroscopy, particularly when combined with spin-trapping techniques, has emerged as a powerful and sensitive analytical method to detect and characterize ROS radicals in biological systems [30,31]. ESR spectroscopy facilitates the direct identification of transient radicals by stabilizing them using spin-trapping agents, including 5-ethoxycarbonyl-5-methyl-1-pyrroline-N-oxide (EMPO) [32,33] and nitroxyl radical probes such as 4-trimethylammonium-2,2,6,6-tetramethylpiperidine-1-oxyl (CAT-1) [34,35]. These advanced analytical tools provide robust, non-invasive means to investigate ROS dynamics, kinetics, and mechanistic roles during platelet activation, significantly advancing our understanding of their physiological and pathological significance [36,37].

This study specifically aims to elucidate the role of ROS, with a particular focus on carbon-centered radicals, in collagen-induced platelet activation. By employing ESR spectroscopy combined with spin-trapping methodologies, this investigation intends to clarify the detailed mechanisms by which ROS generation contributes to platelet aggregation and oxidative stress. Additionally, we explore the potential protective effects of various antioxidants, both enzymatic (superoxide dismutase and catalase) and non-enzymatic (DMTU, Tiron, and mannitol), in attenuating oxidative damage induced by collagen stimulation. The findings derived from this study may offer novel insights into platelet biology, potentially leading to innovative therapeutic strategies for preventing thrombotic disorders and managing cardiovascular disease risk.

## 2. Materials and Methods

### 2.1. Reagents

The structures and abbreviations of the nitroxyl probe and spin-trapping agent used are shown in Table 1 [38,39]. CAT-1 iodide was purchased from Molecular Probes, Inc. (Eugene, OR, USA) and 2-Ethoxycarbonyl-2-methyl-3,4-dihydro-2H-pyrroline-1-oxide (EMPO) was purchased from Oxis International (Portland, OR, USA). The nitroxyl probe was dissolved in isotonic phosphate-buffered saline (50 mmol/L sodium phosphate buffer containing 0.67 *w*/*v* % NaCl, pH 7.4) at the concentrations indicated and passed through a membrane filter (pore size, 0.2 μm). Collagen (Type I) was obtained from Chrono-Log Corporation (Havertown, PA, USA). Dimethyl thiourea (DMTU), D (-)-mannitol, Cu/Zn superoxide dismutase (SOD) from bovine erythrocytes, catalase from bovine liver, and Tiron were sourced from Sigma Chemical Co., (St. Louis, MO, USA). All other reagents used were of the highest purity commercially available.

### 2.2. Animals

Male ICR mice (3.5 weeks old, body weight range 18–22 g) were obtained from Seac Yoshitomi, Ltd. (Fukuoka, Japan). Upon arrival, the mice underwent a 1-week acclimation period to adapt to laboratory conditions prior to experimentation. Animals were housed in standard polycarbonate cages under controlled environmental conditions, specifically maintained at a temperature of 23 ± 2 °C and a relative humidity of 50%, with a consistent 12 h light/dark cycle (lights on from 07:00 to 19:00).

Throughout the duration of the experimental period, the mice were provided with unrestricted access to a standardized commercial rodent diet (MF, Oriental Yeast Co., Ltd., Tokyo, Japan), formulated to meet nutritional requirements. Drinking water was continuously supplied *ad libitum* via sterilized water bottles with daily replenishment and routine checks to ensure cleanliness and availability. All protocols and procedures conducted in this study received ethical review and formal approval from the Institutional Animal Care and Use Committee (IACUC) of Kyushu University, under ethical approval number A22-054-1, officially granted on 1 April 2010.

### 2.3. Preparation of Platelet-Rich Plasma (PRP)

With approval from the Institutional Review Board of Seoul National University (IRB No. 1702/003-004, 5 March 2019), whole blood was collected from healthy male volunteers aged between 18 and 25 years, who were non-smokers and had abstained from medication or alcohol intake for at least 72 h prior to the experiment. Blood samples were carefully drawn into sterile vacutainer tubes containing 3.8% sodium citrate solution to serve as an anticoagulant, preventing blood coagulation during handling. All blood collection procedures were performed gently to avoid platelet activation by minimizing exposure to strong mechanical forces and ensuring the use of plastic rather than glass materials.

The collected blood samples were immediately processed to prepare platelet-rich plasma (PRP) by centrifugation at a gentle force of 100× *g* for 10 min at room temperature. Following this initial centrifugation step, the platelet-rich supernatant was carefully transferred to a fresh polypropylene tube. Platelet-poor plasma (PPP), serving as a control and baseline reference, was prepared by subjecting an aliquot of the PRP to a second centrifugation step at 300× *g* for 20 min, as described by Chung et al. [40]. The platelet concentration in PRP was standardized to a final density of approximately 3 × 10^8^ platelets/mL by dilution with PPP.

### 2.4. Measurement of Platelet Aggregation

Platelet aggregation was measured turbidometrically utilizing a Lumi-aggregometer (Chrono-Log Corp., Havertown, PA, USA) equipped with a temperature-controlled cuvette holder set at 37 °C. PRP samples (500 μL each) were pre-warmed and stirred gently at a constant speed of 1200 rpm for precisely 1 min to ensure optimal mixing and uniform temperature before the addition of the agonist. After the equilibration period, collagen was introduced into the PRP samples at varying concentrations ranging from 0 to 2 μg/mL to initiate platelet aggregation. Aggregation was continuously monitored by measuring changes in light transmission for a duration of 10 min after collagen addition.

### 2.5. ESR Measurement

Anesthesia was induced via an intramuscular injection of urethane at a dose of 1.8 g/kg body weight. A sterilized solution of each probe in PBS (100 μL) was instilled into the blood of the anesthetized mice. For intravenous (i.v.) injection, a sterilized 50 mmol/L solution of each probe in PBS (50 μL) was injected via the tail vein of the anesthetized mouse.

In vivo ESR spectra were recorded immediately at the mid-thorax using a JEOL JES PE-1X spectrometer equipped with a JEOL L-band ESR unit (ES-LBIA) and a loop-gap resonator (33 mm i.d. × 5 mm long). The microwave frequency was 1.1 GHz, with a power setting of 5.0 mW. Field modulation at 100 kHz had an amplitude of 0.16 mT. The external magnetic field was swept at a rate of 5 mT/min, and spectra were recorded repeatedly. The rates of signal decay were obtained from semi-logarithmic plots of the signal intensities. Collagen was injected intravenously, followed by 100 μL of 50 mM CAT-1 in PBS administered 1 min later. Antioxidants (SOD, catalase, Tiron, and hydroxyl radical scavengers) were co-administered with CAT-1 in selected experiments.

In vitro ESR spectra were recorded using a JEOL JES RE-1X spectrometer (JEOL Ltd., Tokyo, Japan). The operating parameters were microwave frequency, 9.4 GHz; microwave power, 7 mW; modulation frequency, 100 kHz; modulation amplitude, 0.7 G; magnetic field center, 336 mT; scan rate, 5 mT/2 min. A reaction mixture (100 μL) containing PRP and 25 mM EMPO was incubated for 2 min before collagen addition. A total of 5 successive scans were averaged for each EMPO spectrum to enhance the signal-to-noise ratio.

### 2.6. Survival Assay Method

Survival rates were determined following the intravenous injection of collagen (0.75 μg/g body weight) into the tail vein of mice. Various antioxidants, including DMTU (3 μM/mouse), mannitol (3 μM/mouse), Tiron (4 μM/mouse), SOD (300 U/mouse), and catalase (100 U/mouse), were administered at various time points prior to collagen injection. Mouse survival was recorded and expressed as the percentage of surviving mice relative to the total number of animals per group.

### 2.7. Statistical Analysis

All values are expressed as mean ± S.D. Data were statistically analyzed using a Tukey–Kramer test or Student’s *t*-test.

## 3. Results

### 3.1. Dose-Dependent Effects of Collagen on Platelet Aggregation and EMPO Adduct Formation In Vitro

**Figure 1:**
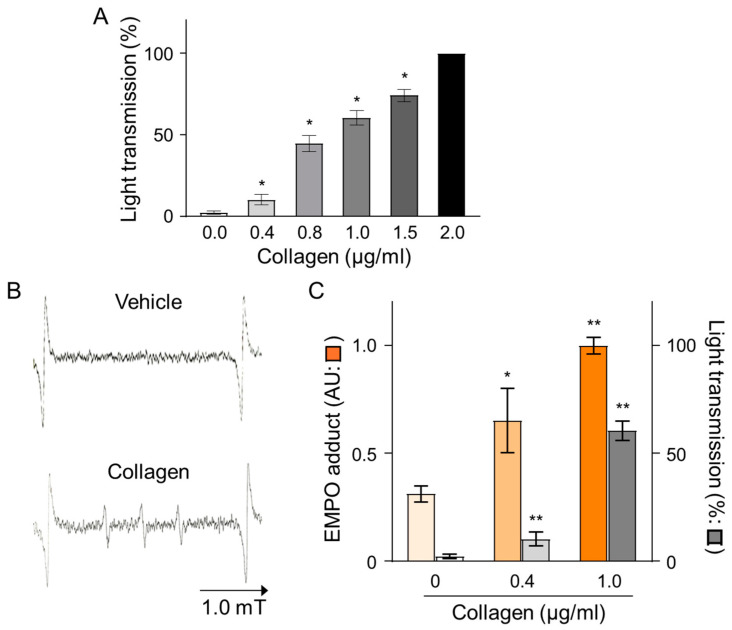
Dose-dependent effects of collagen treatment on EMPO adducts under collagen-induced platelet aggregation in human PRP. (**A**) Collagen (0–2 μg/mL) was added to PRP, and platelet aggregation was calculated as a percentage relative to vehicle-treated controls (0.9% NaCl, 0 μg/mL). Data are presented as mean ± SD (*n* = 4–6). (**B**) Representative ESR spectra of EMPO adducts formed in human PRP treated with collagen (1 μg/mL) or vehicle (0 μg/mL). PRP was pre-incubated with 25 mM EMPO for 2 min before collagen addition, and spectra were recorded 2 min post-treatment. (**C**) Dose-dependent increases in EMPO adduct formation (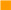) and platelet aggregation (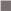) in response to collagen (0–1 μg/mL). Statistical significance was analyzed using Student’s *t*-test with * *p* < 0.005 and ** *p* < 0.01 compared to the vehicle group.

The effects of collagen on platelet aggregation and reactive oxygen species (ROS) generation were evaluated by measuring changes in light transmission and EMPO adduct formation in platelet-rich plasma (PRP) isolated from human volunteers. Treatment with collagen induced a concentration-dependent increase in platelet aggregation, reflected by elevated light transmission values from baseline (0.0 µg/mL) to maximal levels observed at the highest collagen concentration (2.0 µg/mL), indicating nearly complete platelet aggregation (Figure 1A). Specifically, collagen concentrations of 0.4, 0.8, 1.0, and 1.5 µg/mL significantly enhanced aggregation in a dose-dependent manner, as reflected by statistically significant incremental increases (* *p* < 0.05). Furthermore, the ROS generation induced by collagen treatment was clearly evidenced by electron spin resonance (ESR) spectroscopy utilizing EMPO spin-trapping techniques. ESR spectra showed a distinct EMPO adduct signal formation in collagen-treated samples compared to negligible signals in vehicle-treated controls, confirming ROS production triggered by collagen exposure (Figure 1B). Quantitative analysis revealed a clear dose-dependent increase in EMPO adduct formation, correlating closely with the observed platelet aggregation responses. Notably, collagen at 1.0 µg/mL induced maximal EMPO adduct formation, paralleling the observed peak aggregation responses (Figure 1C). These findings strongly indicate a direct relationship between platelet aggregation and oxidative stress over the collagen concentrations applied, emphasizing that collagen-induced ROS generation significantly contributes to platelet activation and subsequent aggregation processes.

### 3.2. Effect of Collagen on CAT-1 Decay and ROS Generation In Vivo

**Figure 2:**
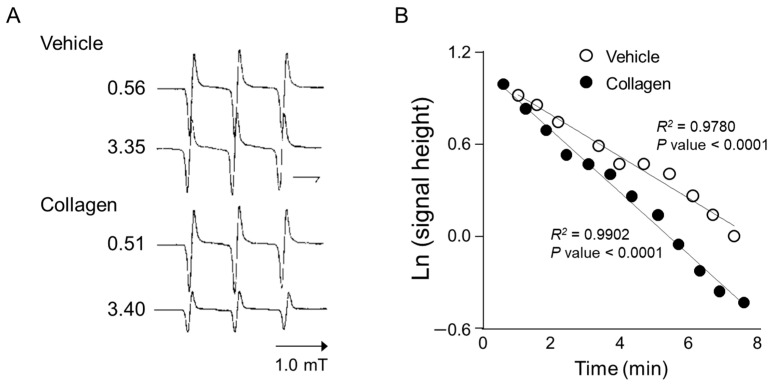
ESR spectrum of CAT-1 detected at the mid-thorax of a collagen-treated mouse. (**A**) Representative L-band ESR spectra recorded at the mid-thorax of an anesthetized mouse 1 min after injection of collagen (i.v. 0.75 μg/g body weight). Subsequently, 100 μL of 10 mM CAT-1 solution was administered via tail vein. (**B**) Decay of CAT-1 ESR signal in collagen- (●) and vehicle-treated (○) mice. Collagen-treated mice received 0.75 μg/g body weight of collagen intravenously, while vehicle-treated mice were administered 0.9% NaCl. Signal intensities at the low-field peak were logarithmically plotted against time post-CAT-1 injection.

Anesthetized mice received intravenous administration of the nitroxyl radical probe CAT-1 (4-trimethylammonium-2,2,6,6-tetramethylpiperidine-1-oxyl), and electron spin resonance (ESR) spectra were recorded in vivo immediately after administration to assess reactive oxygen species (ROS) generation. Representative ESR spectra from vehicle-treated controls showed relatively stable and higher signal intensities at both early (0.56 min) and later time points (3.35 min), indicating minimal ROS generation and slower CAT-1 decay (Figure 2A, Vehicle). In contrast, mice treated with collagen exhibited a notable decrease in ESR signal intensity at the corresponding time points, demonstrating substantially higher ROS generation and faster CAT-1 decay rates (Figure 2A, collagen). Specifically, collagen administration led to a pronounced reduction in ESR signal intensities, indicating accelerated ROS-mediated oxidation and the rapid clearance of the nitroxyl radical probe from circulation.

Quantitative kinetic analysis further supported these observations by clearly demonstrating a significant acceleration in CAT-1 decay kinetics in collagen-treated mice compared to vehicle-treated controls (Figure 2B). Linear regression analysis showed a strong linear relationship (*R*^2^ > 0.97; *p* < 0.0001 for both groups), indicating first-order decay kinetics. The collagen-treated mice displayed a significantly faster decay rate (slope: −0.203) compared to the controls (slope: −0.137), confirming enhanced oxidative stress in response to collagen administration. These detailed kinetic analyses strongly confirm that collagen significantly accelerates ROS generation and nitroxyl radical probe decay in vivo, providing clear evidence of collagen’s capacity to induce oxidative stress linked to platelet activation and thrombosis.

### 3.3. Time-Course Analysis

**Figure 3:**
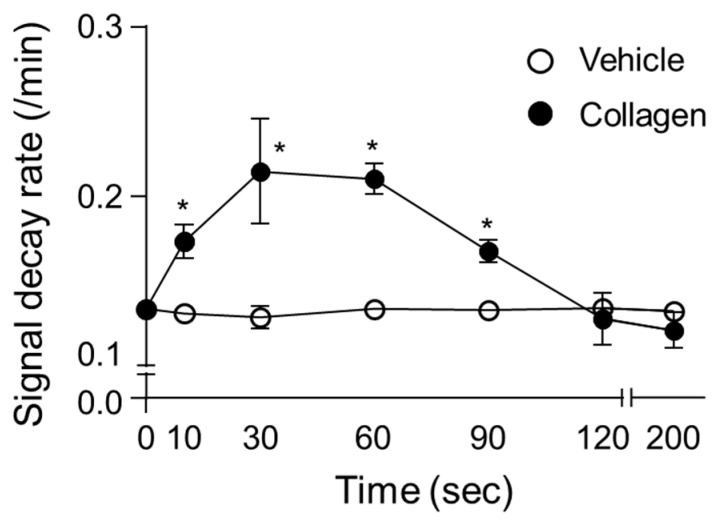
Time-dependent changes in decay rate of CAT-1 in collagen-treated mice. Collagen (●, 0.75 μg/g body weight) or vehicle (○, 0.9% NaCl) was injected intravenously into mice, followed by administration of 100 μL of 10 mM CAT-1 solution. CAT-1 decay rates were determined using L-band ESR spectroscopy at various times post-collagen administration. Data are presented as mean ± SD (*n* = 6–11). * Indicates significant differences between collagen- and vehicle-treated mice (*p* < 0.005, Student’s *t*-test).

The time-course analysis of the CAT-1 decay rate demonstrated significant temporal variations over the measurement intervals following collagen administration in vivo. In collagen-treated mice, the decay rate of the ESR signal rapidly increased from baseline (0 min), reaching a significant peak between 30 s and 1 min post-administration (Figure 3). Specifically, the decay rate sharply rose from the initial measurement (baseline at 10 s) to a peak at approximately 60 s, displaying significantly elevated ROS activity compared to the vehicle-treated control group. After reaching peak levels, the ROS generation gradually decreased over subsequent intervals (90 and 120 s) yet remained above baseline levels, before returning closer to control levels at 200 s post-administration. Conversely, the vehicle-treated mice showed minimal fluctuation in ROS levels throughout the measurement period, reflecting consistently low and stable decay rates. This clear transient pattern of ROS generation after collagen exposure highlights a rapid and dynamic oxidative response, indicative of acute, yet temporary oxidative stress induction. The high consistency and reproducibility of these observations across multiple replicates further emphasize the reliability and physiological relevance of collagen-induced ROS generation patterns in vivo (Figure 3).

### 3.4. Impact of Increasing Collagen Concentrations on ROS Generation and Survival in Mice

**Figure 4:**
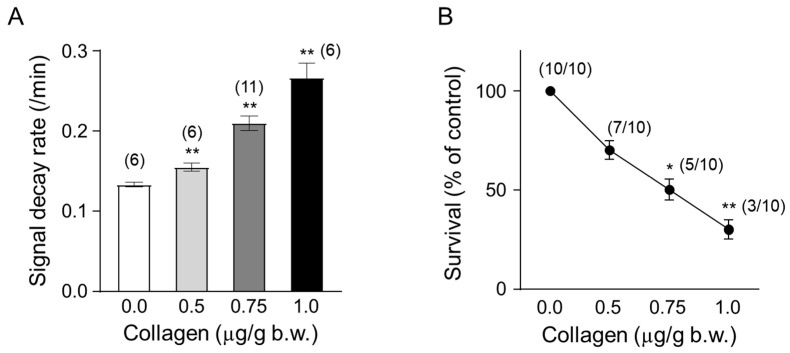
Temporal changes in collagen content. (**A**) Dose-dependent effect of collagen on the CAT-1 decay rate. One minute after the indicated collagen doses, 100 μL of 10 mM CAT-1 solution was administered intravenously, and decay rates were recorded using L-band ESR spectroscopy. Vehicle-treated mice (0 μg collagen) received 0.9% NaCl. (**B**) Survival rate of collagen-treated mice. Numbers in parentheses indicate the number of mice surviving per total number tested. Data are presented as mean ± SD (*n* = 6–11); * and ** indicate significant differences compared to vehicle-treated mice (*p* < 0.01, *p* < 0.005, respectively, Student’s *t*-test).

Mice were administered collagen at increasing concentrations (0.5, 0.75, and 1.0 µg/g body weight) to evaluate the dose-dependent effects on ROS generation and survival rates in vivo. Collagen administration led to a clear dose-dependent increase in ROS generation, measured by the accelerated decay rate of the ESR signal intensity of the nitroxyl radical probe CAT-1 (Figure 4A). Specifically, the CAT-1 signal decay rate significantly increased at 0.5 µg/g collagen (approximately 0.155/min, ** *p* < 0.01) and was further enhanced at 0.75 µg/g (approximately 0.210/min; ** *p* < 0.01) and reached the highest level at 1.0 µg/g body weight (approximately 0.267/min, ** *p* < 0.01). These results indicated robust ROS induction associated directly with increased collagen concentrations.

In parallel, the survival rates of collagen-treated mice significantly decreased in a dose-dependent manner (Figure 4B). While all mice (10/10, 100%) survived following administration of the lowest collagen concentration, survival progressively diminished with increasing doses. Survival rates decreased to 70% (7/10) at 0.5 µg/g collagen (* *p* < 0.05), further declining to approximately 50% (5/10 mice) at 0.75 µg/g body weight (* *p* < 0.05) and ultimately reached a significantly lower survival rate of about 30% (3/10 survival, ** *p* < 0.01) at the highest collagen concentration tested (1.0 µg/g body weight). These findings strongly demonstrate a direct correlation between collagen-induced ROS generation intensity and reduced animal survival, underscoring the critical physiological implications and potential risks associated with elevated collagen-mediated oxidative stress and thrombosis.

### 3.5. Effects of Antioxidants on Collagen-Induced Oxidative Stress and Animal Survival

**Figure 5:**
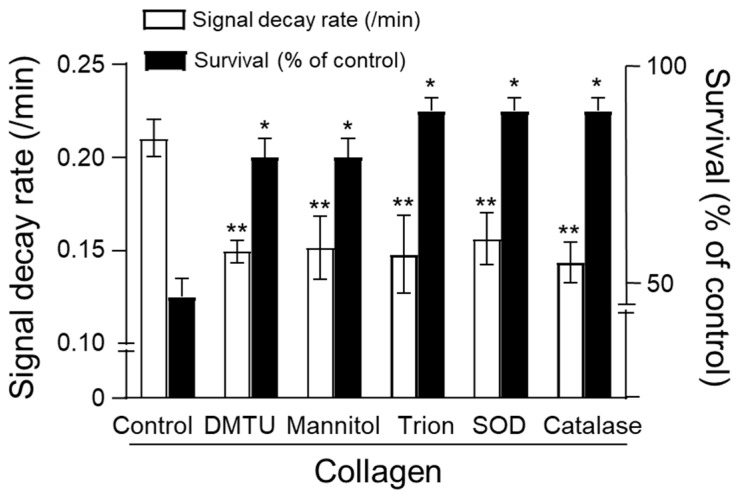
Effect of antioxidants on survival. Collagen (0.75 μg/g body weight) was injected intravenously, followed by 100 μL of 10 mM CAT-1 solution. ESR spectra recorded 1 min post-CAT-1 injection. Antioxidants, including DMTU (3 μM/mouse), mannitol (3 μM/mouse), Tiron (4 μM/mouse), SOD (300 U/mouse), and catalase (100 U/mouse), were administered at various times relative to collagen injection. Survival rates (■) and CAT-1 decay rates (□) are expressed as percentages compared to collagen-only controls. * Indicates significant differences in survival (*p* < 0.01), and ** indicates significant differences in decay rates (*p* < 0.005, Student’s *t*-test).

The protective effects of enzymatic and non-enzymatic antioxidants against collagen-induced oxidative stress were assessed by measuring CAT-1 decay rates and survival in mice. Collagen administration alone significantly increased the CAT-1 decay rate, indicating enhanced ROS generation, and substantially reduced survival rates compared to vehicle-treated controls (Figure 5). Treatment with non-enzymatic antioxidants, including dimethylthiourea (DMTU), mannitol, and Tiron, significantly attenuated the collagen-induced increase in CAT-1 decay rates, demonstrating effective ROS scavenging (DMTU: ~0.14/min, Mannitol: ~0.15/min, Tiron: ~0.14/min; ** *p* < 0.01 compared to collagen alone). Similarly, enzymatic antioxidants, superoxide dismutase (SOD) and catalase, also significantly reduced CAT-1 decay rates (SOD: ~0.15/min; catalase: ~0.14/min; ** *p* < 0.01).

Importantly, these antioxidants markedly improved animal survival following collagen challenge. Mice receiving antioxidant treatments showed significantly higher survival rates (approximately 85% to 90%; * *p* < 0.05 compared to the collagen-treated group) relative to the collagen-only group, which exhibited a dramatically reduced survival rate of about 48%. These results collectively highlight the efficacy of both enzymatic and non-enzymatic antioxidants in mitigating collagen-induced ROS-mediated oxidative damage, underscoring their potential therapeutic value in managing thrombotic disorders associated with oxidative stress.

## 4. Discussion

**Figure 6:**
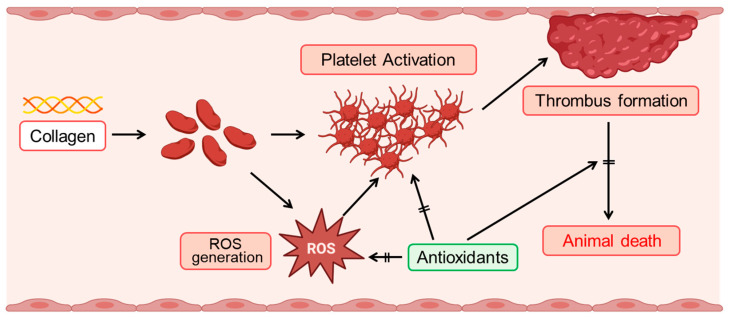
Schematic illustration of collagen-induced ROS production and platelet activation, and the protective role of antioxidants. Collagen exposure induces reactive oxygen species (ROS) generation, which subsequently triggers platelet activation. Activated platelets aggregate, facilitating thrombus formation. Enhanced thrombus formation can ultimately lead to animal death. Antioxidants effectively mitigate these adverse effects by suppressing ROS generation, thereby inhibiting platelet activation, reducing thrombus formation, and improving survival outcomes.

Our findings elucidate the significant role of collagen in modulating oxidative stress and platelet function. Collagen exposure enhanced reactive oxygen species (ROS) generation, altered CAT-1 decay kinetics, and induced dose-dependent platelet aggregation, reinforcing its critical involvement in vascular pathology and thrombosis (Figure 6). The increased ROS generation observed in collagen-treated groups (0.5–1 μg/g body weight) underscores collagen’s pro-oxidative nature (Figure 4A). The significant reduction in electron spin resonance (ESR) signal intensity, coupled with the accelerated CAT-1 decay rates, robustly supports collagen’s role in stimulating ROS production, consistent with its known ability to enhance platelet activation [5]. Our observations of first-order decay kinetics align with previous studies highlighting extracellular matrix components, particularly collagen, in oxidative stress processes [41].

The time-course analysis provided crucial insights into the dynamic nature of ROS activity, peaking rapidly at 0.5–1 min post-collagen administration (Figure 3). This transient oxidative burst aligns well with ROS-mediated kinetic responses documented previously [42]. The subsequent decline toward baseline likely reflects adaptive antioxidant responses or the consumption of ROS scavengers, highlighting the transient yet impactful nature of oxidative stress during acute thrombotic events [43].

Mechanistically, our data suggest that collagen promotes carbon-centered radical formation via the arachidonic acid metabolic pathway, involving fatty acid hydroperoxide decomposition [44]. While our findings primarily support lipid peroxidation pathways as a major source of ROS, NADPH oxidase (NOX), particularly NOX2, may also significantly contribute to collagen-induced oxidative stress. Future studies utilizing specific NOX inhibitors and superoxide-detecting probes are warranted to dissect the relative contributions of each pathway. The formation of these radical intermediates, along with oxygen-centered radicals, likely synergistically enhances ROS generation and subsequent platelet activation. This mechanistic understanding strengthens our hypothesis regarding collagen-induced oxidative stress and lipid peroxidation, establishing a foundation for further mechanistic exploration [45].

The dose-dependent effect of collagen on platelet aggregation highlights a nuanced threshold response. Moderate collagen concentrations (~0.4 μg/mL) induced intermediate platelet aggregation, whereas higher concentrations (≥1 μg/mL) reached a plateau (Figure 1C), suggesting potential regulatory feedback mechanisms at elevated collagen exposure. These results underscore the importance of optimal collagen concentrations in modulating thrombotic responses, supporting findings from Sharma et al. [46].

Antioxidant treatments, particularly enzymatic antioxidants such as superoxide dismutase (SOD) and catalase, substantially mitigated collagen-induced oxidative stress, significantly enhancing survival rates (Figure 5) [47,48]. Additionally, non-enzymatic antioxidants (DMTU, Tiron, and mannitol) demonstrated similar protective effects, suggesting their broader therapeutic potential [49]. These observations highlight the importance of targeting superoxide and hydrogen peroxide pathways to attenuate ROS-mediated cellular damage.

Moreover, our findings suggest potential clinical implications for antioxidant therapy in managing thrombotic disorders associated with elevated oxidative stress. Therapeutic antioxidant strategies may include pharmacologic SOD mimetics (e.g., Tempol), catalase-like compounds, or NOX inhibitors. Combining these agents with conventional anti-platelet therapies may offer a synergistic approach to reduce oxidative stress-induced thrombotic risk. Specifically, we describe the potential for targeting superoxide and hydrogen peroxide pathways via SOD mimetics or catalase enhancers, as well as the possibility of combining antioxidant therapy with conventional anti-platelet agents for synergistic effect. The protective efficacy observed with both enzymatic and non-enzymatic antioxidants underscores their therapeutic relevance in preventing platelet hyperactivation and thrombus formation. Given the critical role of oxidative mechanisms in cardiovascular diseases, including myocardial infarction, stroke, and atherosclerosis [7], further studies are necessary to determine optimal antioxidant dosing, targeted delivery, and long-term safety. Although the current study did not directly evaluate the combined effects of antioxidants and conventional antithrombotic agents, we hypothesize that such combinations may confer enhanced protection against collagen-induced oxidative stress and thrombosis. Thus, future studies investigating these potential synergistic effects are critical for developing improved therapeutic strategies for thrombotic disorders.

Our study further emphasizes the critical interplay between ROS generation and platelet aggregation induced by collagen exposure. Specifically, the concentration-dependent increase in platelet aggregation, evidenced by enhanced light transmission in platelet-rich plasma (PRP), underscores collagen’s pivotal physiological role in platelet activation.

ESR analysis utilizing EMPO spin-trapping clearly demonstrated that collagen treatment robustly induced ROS production, further linking oxidative stress directly to platelet activation processes. The peak aggregation and ROS generation at intermediate collagen concentrations (1 μg/mL) observed in vitro suggest tightly regulated mechanisms maintaining oxidative and thrombotic balance (Figure 1C).

Complementarily, in vivo analysis demonstrated a rapid and marked increase in ROS generation, indicated by significantly accelerated CAT-1 decay rates immediately following collagen administration. These rapid, transient oxidative responses closely mirror pathophysiological events observed in thrombotic diseases, emphasizing the brief yet intense nature of oxidative stress during vascular injury responses.

In addition, our survival data clearly delineate a dose-dependent negative impact of collagen-induced ROS on animal survival, emphasizing the potential clinical implications of elevated oxidative stress levels. The observed protective effects of antioxidants further highlight ROS as promising therapeutic targets for mitigating platelet hyperactivation and associated thrombotic complications.

Collectively, our comprehensive investigation clarifies the mechanistic relationships between oxidative stress and platelet function induced by collagen, providing substantial evidence to support targeted antioxidant strategies for preventing or reducing thrombotic risks in cardiovascular disorders.

## 5. Conclusions

This study provides significant insights into the interplay between collagen-induced oxidative stress and platelet activation, highlighting potential therapeutic opportunities for antioxidants in managing thrombotic and cardiovascular risks.

## Figures and Tables

**Table 1:** Nitroxyl spin probes and spin-trapping agents used.

Abbreviation	Structure	*K*_p_* ^†^
CAT-1	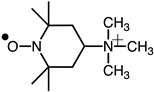	0.0033 *^1^
EMPO		<0.15 *^2^
	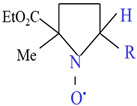	

^†^ 1-Octanol/isotonic buffered solution (pH 7.1–7.4) partition coefficient. *^1^: Ref Takeshita et al. [38], *^2^: Ref Stolze et al. [39].

## Data Availability

The data presented in this study are available upon request from the corresponding author.

## Abbreviations

PRP: platelet-rich plasma; ESR: electron spin resonance; CAT-1: 4-trimethylammonium-2,2,6,6-tetramethylpiperidine-1-oxyl; EMPO: 5-ethoxycarbonyl-5-methyl-1-pyrroline N-oxide.
